# Histone deacetylase inhibition is synthetically lethal with arginine deprivation in pancreatic cancers with low argininosuccinate synthetase 1 expression

**DOI:** 10.7150/thno.40195

**Published:** 2020-01-01

**Authors:** Stephanie S. Kim, Shili Xu, Jing Cui, Soumya Poddar, Thuc M. Le, Hovhannes Hayrapetyan, Luyi Li, Nanping Wu, Alexandra M. Moore, Lei Zhou, Alice C. Yu, Amanda M. Dann, Irmina A. Elliott, Evan R. Abt, Woosuk Kim, David W. Dawson, Caius G. Radu, Timothy R. Donahue

**Affiliations:** 1 Department of Surgery, UCLA, Los Angeles, CA, 90095, USA.; 2 Department of Pancreatic Surgery, Union Hospital, Tongji Medical College, Huazhong University of Science and Technology (HUST), Hubei 430022, China; 3 Ahmanson Translational Imaging Division, UCLA, Los Angeles, CA, 90095, USA.; 4 Department of Molecular and Medical Pharmacology, UCLA, Los Angeles, CA, 90095, USA.; 5 Department of Pancreatic and Thyroidal Surgery, Shengjing Hospital, China Medical University, Shenyang 110003, China; 6 Department of Pathology and Laboratory Medicine, UCLA, Los Angeles, CA 90095, USA.; 7 Jonsson Comprehensive Cancer Center, UCLA, Los Angeles, CA, 90095, USA; 8 David Geffen School of Medicine, UCLA, Los Angeles, CA, 90095, USA.

**Keywords:** HDAC inhibitor, arginine deprivation, pancreatic cancer, DNA damage, synthetic lethality

## Abstract

Arginine (Arg) deprivation is a promising therapeutic approach for tumors with low argininosuccinate synthetase 1 (ASS1) expression. However, its efficacy as a single agent therapy needs to be improved as resistance is frequently observed.

**Methods:** A tissue microarray was performed to assess ASS1 expression in surgical specimens of pancreatic ductal adenocarcinoma (PDAC) and its correlation with disease prognosis. An RNA-Seq analysis examined the role of ASS1 in regulating the global gene transcriptome. A high throughput screen of FDA-approved oncology drugs identified synthetic lethality between histone deacetylase (HDAC) inhibitors and Arg deprivation in PDAC cells with low ASS1 expression. We examined HDAC inhibitor panobinostat (PAN) and Arg deprivation in a panel of human PDAC cell lines, in ASS1-high and -knockdown/knockout isogenic models, in both anchorage-dependent and -independent cultures, and in multicellular complex cultures that model the PDAC tumor microenvironment. We examined the effects of combined Arg deprivation and PAN on DNA damage and the protein levels of key DNA repair enzymes. We also evaluated the efficacy of PAN and ADI-PEG20 (an Arg-degrading agent currently in Phase 2 clinical trials) in xenograft models with ASS1-low and -high PDAC tumors.

**Results:** Low ASS1 protein level is a negative prognostic indicator in PDAC. Arg deprivation in ASS1-deficient PDAC cells upregulated asparagine synthetase (ASNS) which redirected aspartate (Asp) from being used for *de novo* nucleotide biosynthesis, thus causing nucleotide insufficiency and impairing cell cycle S-phase progression. Comprehensively validated, HDAC inhibitors and Arg deprivation showed synthetic lethality in ASS1-low PDAC cells. Mechanistically, combined Arg deprivation and HDAC inhibition triggered degradation of a key DNA repair enzyme C-terminal-binding protein interacting protein (CtIP), resulting in DNA damage and apoptosis. In addition, S-phase-retained ASS1-low PDAC cells (due to Arg deprivation) were also sensitized to DNA damage, thus yielding effective cell death. Compared to single agents, the combination of PAN and ADI-PEG20 showed better efficacy in suppressing ASS1-low PDAC tumor growth in mouse xenograft models.

**Conclusion:** The combination of PAN and ADI-PEG20 is a rational translational therapeutic strategy for treating ASS1-low PDAC tumors through synergistic induction of DNA damage.

## Introduction

Despite many advances in cancer therapeutics, pancreatic ductal adenocarcinoma (PDAC) continues to have a poor prognosis with a 5-year survival rate of 9% 1. Due to its frequent resistance to chemotherapies, novel treatment strategies are urgently needed to improve the survival of PDAC patients. Metabolic reprogramming is a hallmark of PDAC and supports tumor progression in a nutrient-poor tumor microenvironment. High PDAC cell demand for amino acids 2, 3 has stimulated the development of amino acid depletion therapies to target this vulnerability. Specifically, arginine (Arg) is critical for metabolic functions of PDAC cells including synthesis of proteins, polyamines, nitric oxide, and other amino acids such as proline and glutamate.

There are three main sources of intracellular Arg. First, it can be synthesized intracellularly by a process that includes Argininosuccinate synthetase 1 (ASS1) as the rate-limiting enzyme in the urea cycle, catalyzing Arg from aspartate (Asp) and citrulline (Cit). It should be noted that ASS1 expression has been reported to be decreased in multiple cancers 4. Second, Arg can be imported from the microenvironment; however, it has been reported to exist in the PDAC microenvironment at low levels 5. Finally, it can be generated from recycled proteins in the lysosome 6, 7 or proteasome 8. Cancer cells with reduced ASS1 expression rely on extracellular or recycled Arg for survival and proliferation, a vulnerability that has been leveraged by pharmacologic Arg deprivation using arginine deiminase (ADI) 9, 10.

ADI-PEG20, a pharmacological Arg-degrading agent that depletes extracellular Arg by converting it to Cit, has been investigated in pre-clinical studies and shown promise in clinical trials for cancers with low ASS1 expression, including melanoma and hepatocellular carcinoma 11. A phase 1/1B trial evaluated ADI-PEG20 in combination with nanoparticle albumin-bound (nab)-paclitaxel and gemcitabine in PDAC and found that this combination was well-tolerated with some patients responding to the treatment 12.

Reduced ASS1 expression is associated with aggressive tumor phenotypes 13-15. There is an inverse correlation between proliferation rate and ASS1 expression in cancer cells 14, 16. Mechanistically, reduction of ASS1 expression promotes cancer cell proliferation by shunting Asp towards pyrimidine biosynthesis 16. ADI-PEG20 treatment in ASS1-low cells also reduces thymidine synthesis 14. These findings encourage further exploration of the biology and vulnerabilities of ASS1-low tumors, with a particular focus on nucleotide metabolism.

ASS1-low cancer cells frequently develop resistance to Arg deprivation monotherapy through genetic alterations and/or metabolic re-wiring, suggesting a need for co-targeting the compensatory mechanisms via rationally designed combination therapy regimens 17-19. In this study, we explored the clinical implications of ASS1 expression in PDAC with immunohistochemistry analyses of PDAC patient tumor samples. Through a transcriptome analysis, we found that Arg deprivation in ASS1-low PDAC cells upregulated asparagine synthetase (ASNS), an enzyme known to utilize the nucleotide biosynthesis substrate Asp for asparagine (Asn) synthesis. The resulting nucleotide insufficiency induced cell cycle S-phase retention in ASS1-low PDAC cells. We then performed an unbiased screen of FDA-approved oncology drugs in an ASS1-deficient PDAC model to identify an effective synthetically lethal combination therapy to treat ASS1-low PDAC. This screen revealed histone deacetylase (HDAC) inhibitors in combination with Arg deprivation. This combination was validated in *in vitro* and* in vivo* ASS1-low PDAC models. Mechanistically, we observed that Arg deprivation and HDAC inhibition synergistically induced DNA damage and degradation of a key DNA repair enzyme C-terminal-binding protein interacting protein (CtIP), resulting in apoptosis. In addition, S-phase-retained ASS1-low PDAC cells were highly sensitive to DNA damage. Our findings provide a rationally designed synthetically lethal translational therapeutic strategy for treating ASS1-low PDAC tumors.

## Experimental Procedures

All work was performed with appropriate institutional review board approvals.

### Immunohistochemistry analysis of PDAC tissue microarray

The PDAC tissue microarray (TMA) has been previously described 20 and was generated from surgical resections performed on treatment-naïve, AJCC stage I/II PDAC at UCLA (N=138) with outcomes obtained from a prospectively maintained clinical database. Following heat-induced antigen retrieval in a vegetable steamer with 10 mM sodium citrate (pH 6.0), immunohistochemistry was performed with anti-ASS1 antibody (1:2000, Santa Cruz Biotechnology sc-365475) and SignalStain Boost IHC detection reagent (Cell Signaling Technology). ASS1 expression was quantified by PDAC pathologists across three representative 1.0 mm cores for each tumor using a semiquantitative histoscore (0-300), which was the product of cytoplasmic staining (0= negative, 1= weak, 2= moderate, 3= strong) and percentage (0-100) of tumor cells staining at that intensity. Each tumor was dichotomized into either ASS1-high or ASS1-low expression groups based on median histoscore. Analyses were performed using IBM SPSS Statistics 25 (Armonk, NY). P-value of less than 0.05 was considered statistically significant.

### Cell culture

Panc1, MiaPaca2, Panc02.03, HS766t, HPAF-II, Suit2, Su8686, Panc03.27, and Panc10.05 cells were purchased from the American Type Culture Collection (ATCC). Primary human cancer-associated fibroblasts were isolated from surgical pancreatic cancer specimens by a previously described outgrowth method under an institutional review board approved protocol 21 and characterized by wild-type KRAS status and α-smooth muscle actin positivity as previously described 22. All cells used for experiments were between passages 3 and 20 and maintained in DMEM+10% FBS+1% Penicillin/ Streptomycin, at 37ºC in 5% CO_2_. The immortalized human pancreatic duct epithelial (HPDE) cell line is a gift from Dr. Ming-Sound Tsao (Ontario Cancer Institute, Toronto, Ontario, Canada). HPDE cells were cultured in keratinocyte-SFM +EGF +bovine pituitary extract (ThermoFisher Scientific). Cells were routinely checked for *Mycoplasma* contamination using MycoAlert kit (Lonza).

### Oncology drug library screen

A library of 133 FDA-approved oncology drugs was provided by the National Cancer Institute (NCI). Drugs were arrayed in polypropylene 384-well plates covering a 7-point concentration range (10 µM, 2.5 µM, 625 nM, 156 nM, 39 nM, 9.8 nM, 2.4 nM). Cells were suspended in RPMI 1640 Medium for SILAC (ThermoFisher Scientific) with 10% dialyzed FBS, 0.3 mM L-Lysine (Sigma), 1mM L-Citrulline (Sigma) with or without 1mM L-Arginine (Sigma) and plated at 1000 cells / well density. After 72 h, 50 µL of CellTiter-Glo reagent diluted 1:4 in PBS was added to each well and luminescence readings were performed using a Synergy H1 Hybrid Reader (Biotek). Each condition was assayed in duplicate (n=2) and % proliferation values were calculated by normalizing experimental wells to plate negative controls and averaging replicate values.

### Gene expression by quantitative real-time PCR

Total RNA was isolated from cells using the NucleoSpin RNA Kit (Clontech). Reverse transcription was performed using the High Capacity cDNA Reverse Transcription kit (Life Technologies). Quantitative PCR was performed using EvaGreen qPCR Master Mix (Lamda Biotech). RNA expression values were normalized to beta-actin control gene and then calculated as relative expression to control. Primers used are reported in Supplementary Table S1.

### Animal studies

All animal studies were approved by the UCLA Animal Research Committee (ARC). 4-6-week-old male NSG mice were injected subcutaneously on bilateral flanks with 1 million MiaPaca2, Patu8988t, or Su8686 cells. 7 days after inoculation, tumors were established and treatment was then initiated. Panobinostat was suspended in a solution with 2% DMSO, 2% Tween-80, and 48% PEG300. ADI-PEG20 was suspended in PBS. Both drugs were delivered through intraperitoneal injection two times per week for three weeks. Tumor size measured by caliper and body weight were monitored twice a week. Mice were sacrificed at the end of treatment and tumors were harvested.

### Statistical analysis

Data are presented as means ± SD with indicated biological replicates. Comparisons of two groups were calculated using indicated unpaired or paired two-tailed Student's t-test and P values less than 0.05 were considered significant. All statistical analyses were performed in Graphpad Prism 6.0, with the exception of the tissue microarray immunohistochemistry data.

## Results

### Low ASS1 expression is associated with worse prognosis in PDAC patients

**Figure 1:**
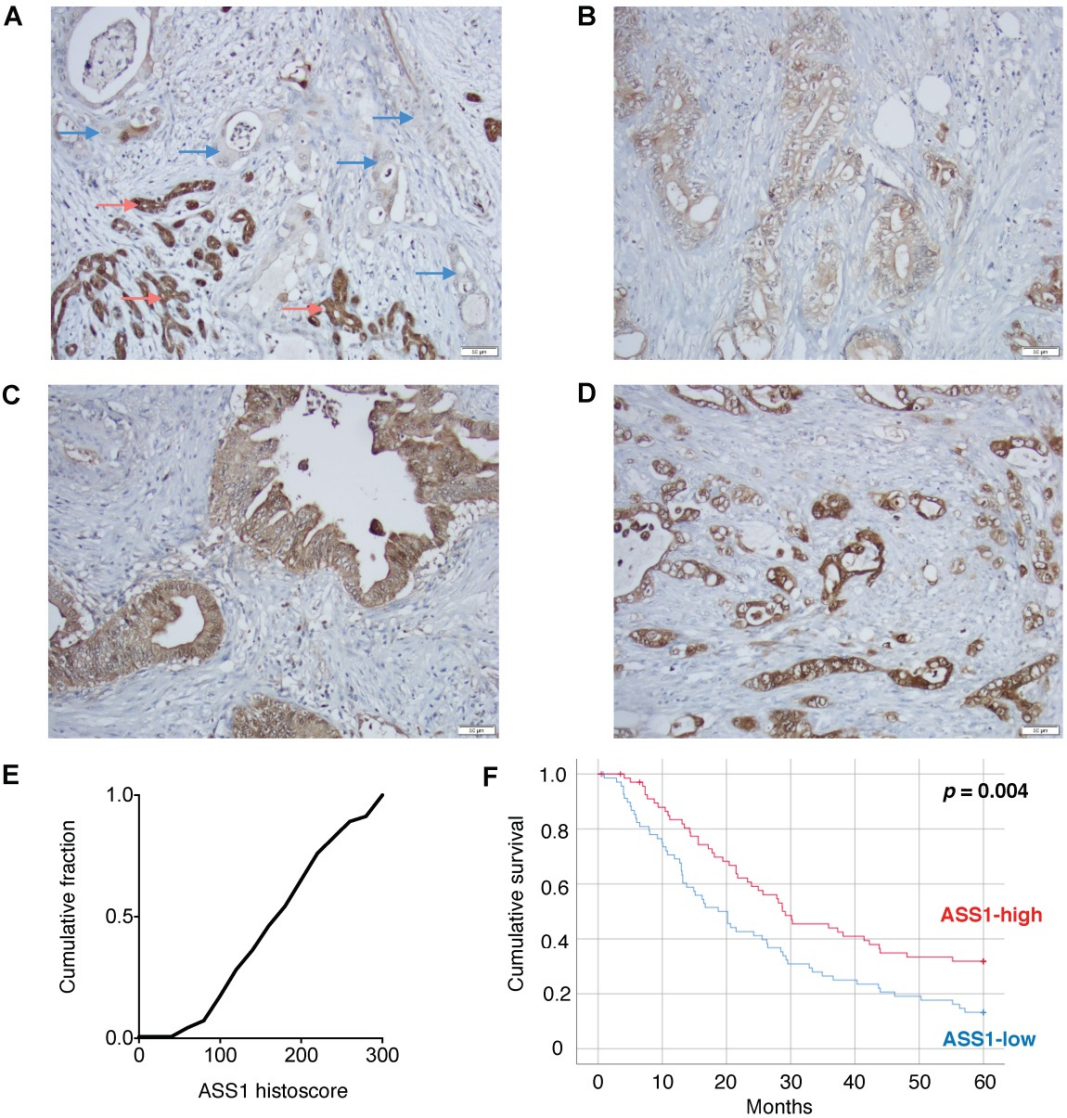
** Low ASS1 expression is associated with worse prognosis in PDAC patients.** (**A**) Absent expression in PDAC cells (blue arrows) infiltrating between benign/reactive ducts with strong ASS1 expression (red arrows). (**B-D**) Representative tumors respectively showing low (1), moderate (2), and strong (3) staining intensity. All images taken using 20x objective (scale bar 50 µM). (**E**) Histoscore distribution of ASS1 expression. Histoscore values represent the sum of tumor cell staining intensity (0-none, 1-weak, 2-moderate, 3-strong) multiplied by the percentage of tumor cells (0-100) at each intensity for a final histoscore range of 0-300. The median histoscore value from three separate cores of a given tumor is depicted and used for analysis. (**F**) Kaplan-Meier analysis of 138 PDAC patients by ASS1 expression.

To assess the clinical importance of ASS1 expression in PDAC, we evaluated ASS1 protein levels in 138 patient tumor samples on a well-annotated PDAC tissue microarray by immunohistochemistry. There was a range of ASS1 protein expression across tumors (Figure 1A-E). We then separated patients into low versus high expression groups based on semiquantitative histoscores. Dichotomized groups were then correlated with clinicopathologic characteristics including gender, age, tumor grade, tumor size, margin status, tumor stage and lymph node status (Supplementary Table S2). Low ASS1 expression was significantly correlated with high-grade histology (p = 0.016), but showed no further significant correlations with gender (p = 0.609), age (p = 0.150), tumor size (p = 0.489), margin status (p = 0.274), AJCC stage (p = 1.00) or lymph node status (p = 1.00). Low ASS1 expression was also significantly correlated with worse overall survival both when patients were dichotomized (p = 0.004, log-rank test) (Figure 1F) or divided based on tertiles (p = 0.031) (Supplementary Figure S1). In addition, low ASS1 expression was an independent predictor of survival in a multivariate Cox regression model with other significant clinicopathologic variables (Supplementary Table S3).

### ASS1 expression in PDAC cell lines inversely correlates with sensitivity to Arg deprivation

**Figure 2:**
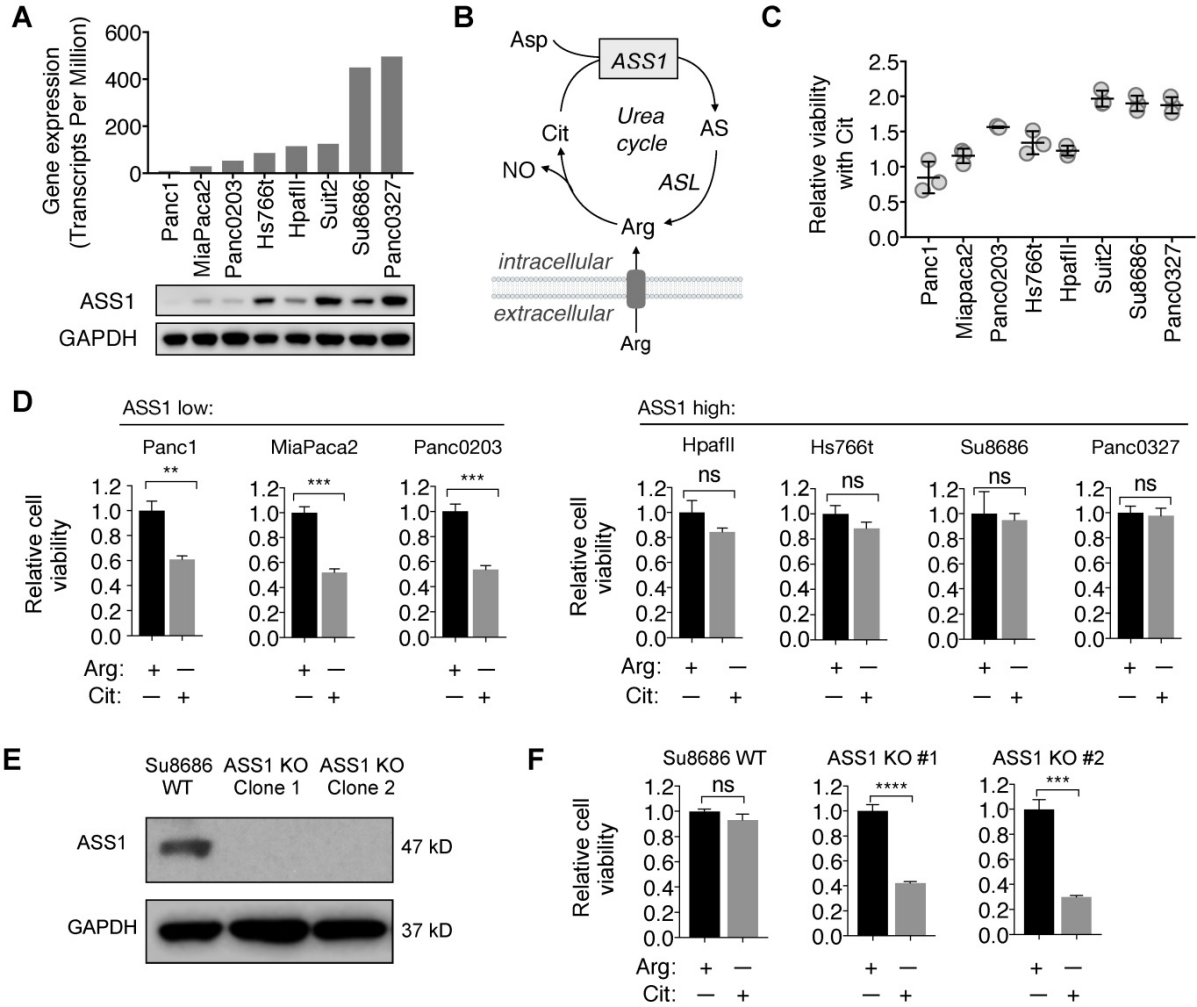
** ASS1 expression in PDAC cells correlates with sensitivity to arginine deprivation.** (**A**) ASS1 mRNA expression on a panel of PDAC cell lines from the Cancer Cell Line Encyclopedia (CCLE) dataset and ASS1 protein expression by western blot on a panel of PDAC cell lines. The average ASS1 mRNA expression was obtained from www.oasis-genomics.org. The immunoblot data represents n=2. (**B**) Reaction catalyzed by ASS1. Aspartate (Asp) and citrulline (Cit) are converted to argininosuccinate (AS) by ASS1 in the urea cycle. AS is then converted to arginine (Arg) by argininosuccinate lyase. NO, nitric oxide. (**C**) Cell viability rescued by Citrulline supplementation, measured by CellTiter Glo. (n=3). (**D**) Cell viability after 72 h Arg deprivation in a panel of PDAC cell lines. Results are shown as mean±SD (n=3). (**E**) Isogenic ASS1 knockout (KO) Su8686 cells generated by CRISPR/Cas9. (**F**) Cell viability with 72 h Arg deprivation and Cit supplementation in Su8686 wild-type (WT) and ASS1 KO cells. (n=3) | RPMI media without Arg and lysine (Lys), supplemented with 0.3 mM Lys and 1 mM Cit ± 1 mM Arg. * p<0.05, ** p<0.01, *** p<0.001, **** p<0.0001. ns, not significant.

Consistent with the immunohistochemistry analysis for ASS1 expression in PDAC tumor samples, there was a range of ASS1 expression in PDAC cell lines at both the mRNA and the protein levels (Figure 2A). We further assessed the activity of ASS1 in these PDAC cell lines by measuring their abilities to use the ASS1 substrate Cit for survival and proliferation in Arg-free cell culture media (Figure 2B). ASS1 expression correlated positively with the degree of cell viability and proliferation with Cit in Arg-free cell culture (Figure 2C). ASS1-low PDAC cells (Panc1, MiaPaca2, Panc0203) were sensitive to Arg deprivation, even in the presence of Cit, whereas ASS1-high PDAC cells (HpafII, Hs766t, Su8686, Panc0327) were resistant to Arg deprivation as they were able to utilize Cit for growth and survival (Figure 2D). To further study the role of ASS1 in PDAC cells, we used CRISPR/Cas9 to knockout ASS1 in an ASS1-high PDAC cell line Su8686 (Figure 2E). In the presence of Arg, ASS1 KO did not affect PDAC cell proliferation. However, in contrast to ASS1-high parental cells, Cit supplementation was not able to rescue isogenic ASS1 knockout (KO) cells in the absence of Arg (Figure 2F). Our results indicate that lack of ASS1 expression renders PDAC sensitive to Arg deprivation, which could not be rescued by Cit.

### Arginine deprivation in ASS1-deficient PDAC cells redirects aspartate from nucleotide *de novo* synthesis

**Figure 3:**
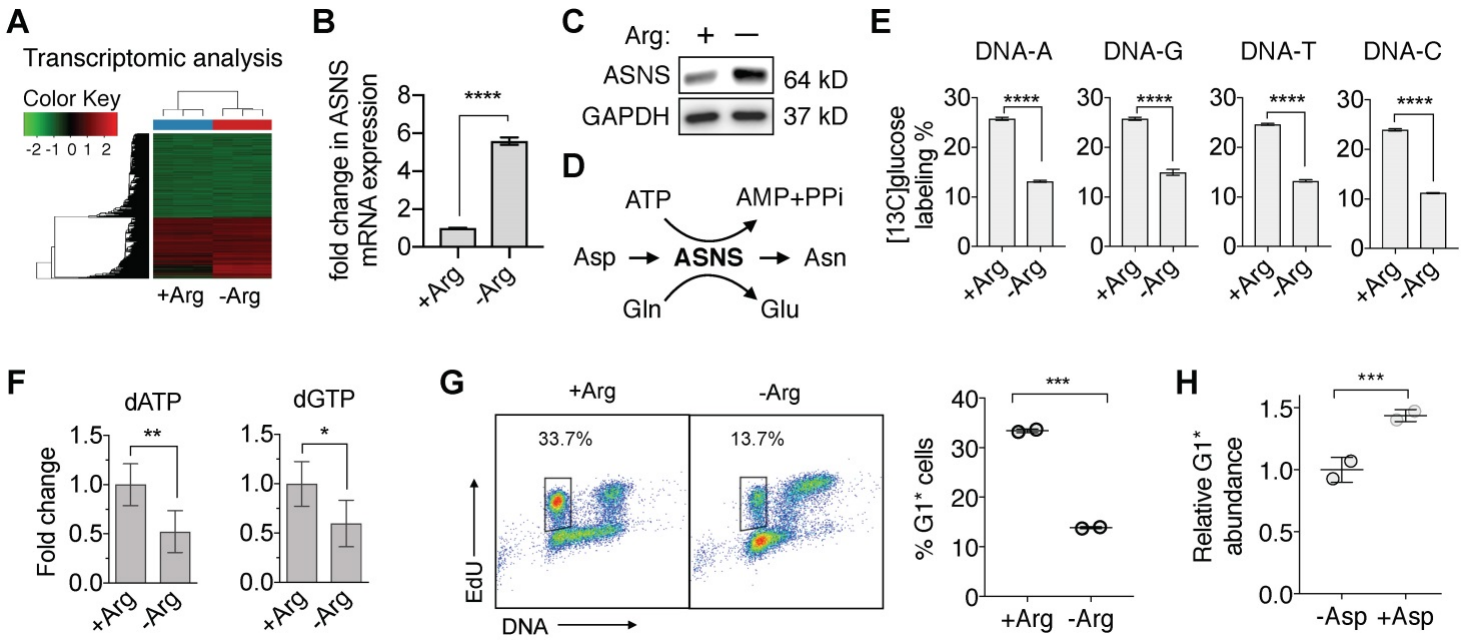
** Arg deprivation re-directs Asp from nucleotide *de novo* synthesis.** (**A**) RNA-seq analysis of Su8686 ASS1 KO cells ± Arg ± PAN for 24 h (n=3). (**B**) Fold change in the mRNA level of asparagine synthetase (ASNS) in Su8686 ASS1 KO cells ± Arg in the RNA-seq analysis. (**C**) ASNS protein expression in Su8686 ASS1 KO cells ± Arg by western blot analysis (24 h). (**D**) Schematic of aspartate (Asp) consumption by ASNS to produce asparagine (Asn). (**E**) LC-MS/MS-MRM analysis of % [^13^C_6_]glucose labeling of DNA after 24 h treatment with ADI-PEG20 (n=3). (**F**) LC-MS/MS-MRM analysis of dATP and dGTP pool fold changes after 48 h Arg deprivation (n=6). (**G**) Arg deprivation induces cell cycle S-phase retention. Flow cytometry analysis of MiaPaca2 cells pulsed for 2 h with EdU to stain S-phase cells, followed by release into media ± Arg for 8 h. Percentage of EdU-labeled cells returning to G1 phase (G1*) is plotted (n=2). (**H**) Measurement of % decrease in G1* cells with Arg deprivation by EdU pulse-chase flow cytometry analysis ± 10 mM Asp in MiaPaca2 cells. | RPMI media without Arg and Lys, supplemented with 0.3 mM Lys and 1 mM Cit ± 1 mM Arg. ADI-PEG20: 0.5 ug/mL. PAN: 50 nM (Su8686 ASS1 KO), 25 nM (MiaPaca2). NT, no treatment, Gln, Glutamine, Glu, Glutamate. * p<0.05, ** p<0.01, *** p<0.001, **** p<0.0001.

To begin to understand the vulnerabilities in ASS1-deficient PDAC cells induced by Arg deprivation, we first performed a comprehensive RNA-sequencing (RNA-Seq) analysis. We identified that in ASS1-deficient PDAC cells, Arg deprivation significantly upregulated transcription of asparagine synthetase (ASNS) (Figure 3A and 3B), which was validated at the protein level (Figure 3C). ASNS converts Asp, an important precursor for *de novo* nucleotide synthesis, to Asn (Figure 3D). We hypothesized that the upregulation of ASNS expression would redirect Asp from being used for *de novo* nucleotide biosynthesis and result in nucleotide insufficiency. To test our hypothesis, we measured both [^13^C_6_]glucose-labeled DNA and total deoxynucleotide pool sizes. Nucleotide *de novo* synthesis was significantly reduced, as indicated by significant reduction in [^13^C_6_]glucose-labeled nucleotides in DNA (Figure 3E) and in dATP and dGTP pool sizes (Figure 3F). Consistent with our previously reported observations 23, 24, nucleotide insufficiency induced by Arg deprivation caused S-phase retention in cell cycle progression (Figure 3G), measured by the percentage of 5-ethynyl-2'-deoxyuridine (EdU)-labeled S-phase cells progressing through mitosis to G1 phase in an EdU-pulse-chase flow cytometric assay 23, 24. Supplementation of Asp in cell culture significantly rescued the cell cycle kinetics (Figure 3H).

### HDAC inhibitors synergize with Arg deprivation to inhibit proliferation of ASS1-deficient PDAC cells

Clinical trials have reported partial response to Arg deprivation therapy with ADI-PEG20 in hepatocellular carcinoma, melanoma, and acute myeloid leukemia, but rates of complete remission were less than 10% in these trials 25-27. While no clinical trial has studied ADI-PEG20 as a single agent therapy in PDAC, a phase I/IB trial with ADI-PEG20, gemcitabine, and nab-paclitaxel showed some promise with disease control rate of 94% 12.

**Figure 4:**
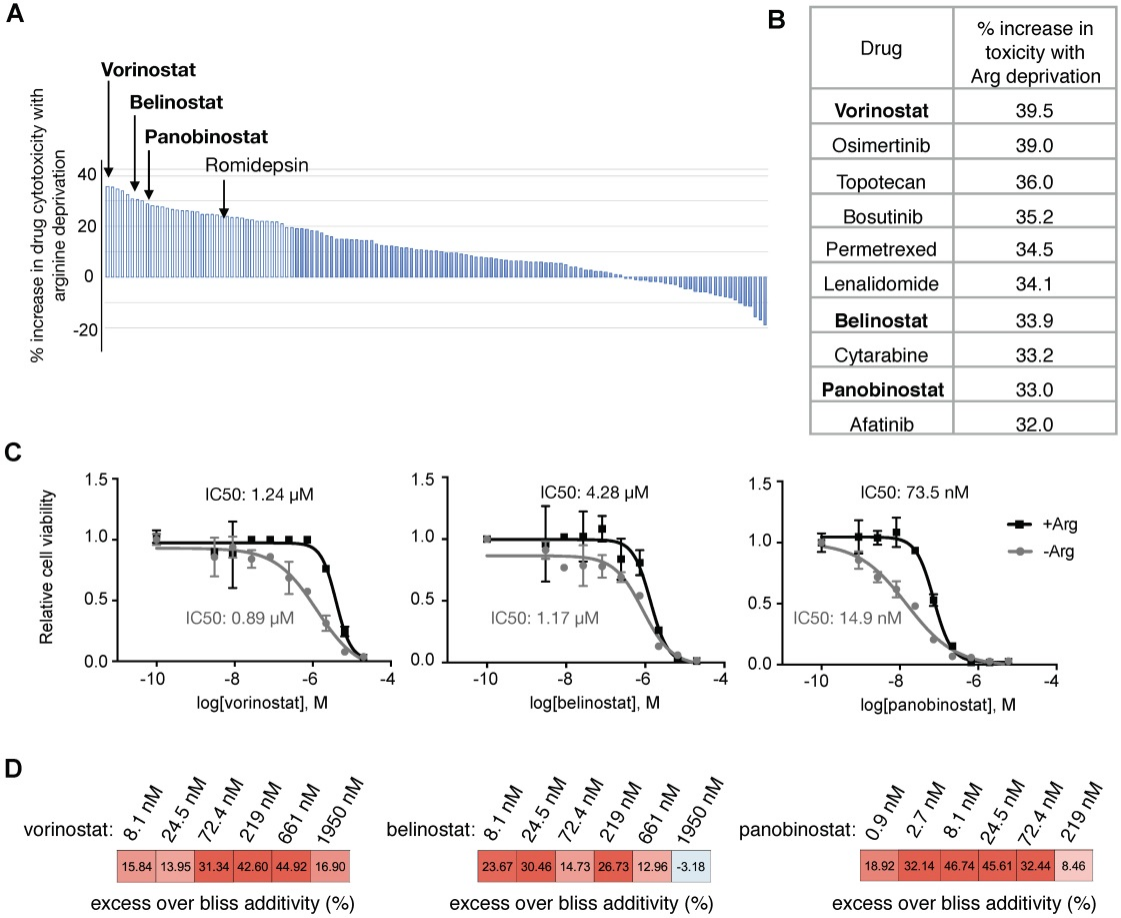
** HDAC inhibitors synergize with Arg deprivation to inhibit ASS1 deficient PDAC cell proliferation.** (**A**) A high throughput screen of 133 FDA-approved oncology drugs identified HDAC inhibitors with increased cytotoxicity in Su8686 ASS1 KO cells in the absence of Arg (n=2). (**B**) Top ten drugs with best synergy with Arg deprivation. (**C**) IC50 values of HDAC inhibitors belinostat, panobinostat, and vorinostat in the presence and absence of Arg (n=3). (**D**) Bliss synergy scores for vorinostat, belinostat, and panobinostat in ASS1-deficient Su8686 cells. Bliss score > 0, synergy. | RPMI media without Arg and Lys, supplemented with 0.3 mM Lys and 1 mM Cit ± 1 mM Arg.

Encouraged by these clinical data with Arg deprivation in combination therapies, we aimed to identify optimal synergistic therapy agents with Arg deprivation in ASS1-deficient PDAC cells. A screen with a library consisting of 133 FDA-approved oncology drugs was performed (Supplementary Table S4). We identified that Arg deprivation sensitized ASS1-deficient PDAC cells to HDAC inhibitors (Figure 4A). Three out of the four HDAC inhibitors in the library, vorinostat, belinostat, and panobinostat were among the top ten drugs that showed enhanced potency with Arg deprivation (Figure 4B). In a secondary screen to validate the HDAC inhibitors, panobinostat showed the greatest decrease in IC50 values with Arg deprivation (Figure 4C). Bliss synergy scores 28 indicated that the interactions between HDAC inhibition and Arg deprivation in ASS1-deficient PDAC cells were synergistic (Figure 4D).

### Low ASS1 expression renders PDAC cells highly sensitive to the combination of arginine deprivation and panobinostat

**Figure 5:**
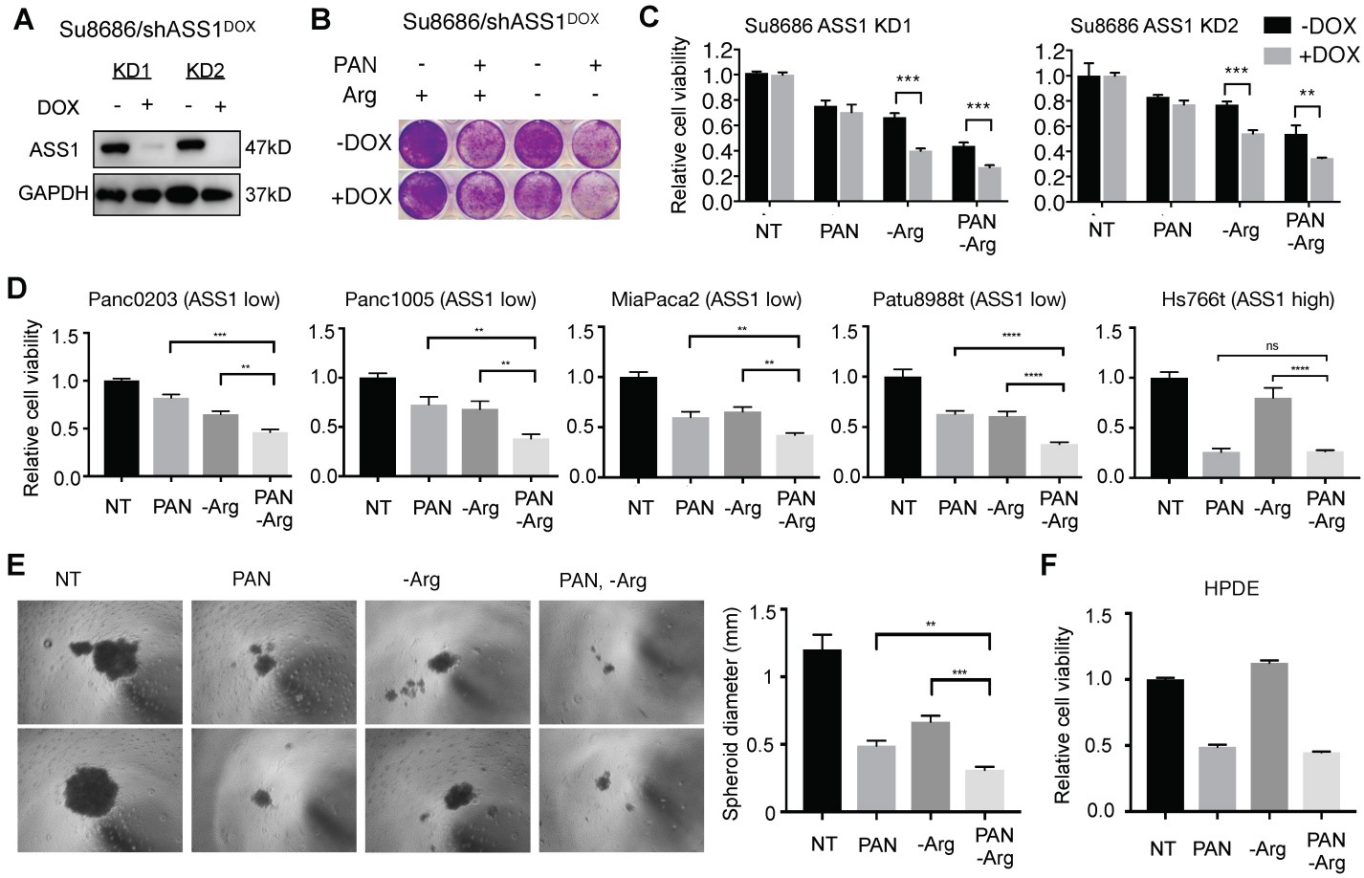
** Low ASS1 expression increases sensitivity to panobinostat and Arg deprivation in ASS1-high Su8686 cells.** (**A**) Doxycycline (DOX)-inducible ASS1 shRNA knockdown (KD) in Su8686 cells. (**B**) Colony formation assay after 7 d treatment with +/- DOX +/- PAN +/- Arg in Su8686 ASS1 KD cells (shRNA 1+2) (n=2). (**C**) Viability of Su8686 cells with and without ASS1 KD exposed to indicated treatments for 72 h. (n=3). (**D**) Viability with 72 h PAN and Arg deprivation treatments in a panel of PDAC cell lines in two dimensional cultures. Results are shown as mean±SD (n=3). (**E**) Patu8988t in anchorage-independent cell culture after 5 d treatment. Left, representative images taken after 5-day treatment. Right, quantification of spheroid diameter. (**F**) Viability with 72 h PAN and Arg deprivation treatment in non-transformed HPDE cells. Results are shown as mean±SD (n=3). | RPMI media without Arg and Lys, supplemented with 0.3 mM Lys and 1 mM Cit ± 1 mM Arg. PAN: 50 nM; DOX: 100 ng/mL. NT, not treated. * p<0.05, ** p<0.01, *** p<0.001, **** p<0.0001.

To further examine the effect of ASS1 expression levels on sensitivity to the combination of Arg deprivation and panobinostat, we established doxycycline (DOX)-inducible shRNA ASS1 knockdown genetic isogenic models of ASS1-high PDAC cell lines Su8686 and Panc0327 (Figure 2A, Figure 5A, and Supplementary Figure S2A). Compared to ASS1-high isogenic cells (-DOX), DOX-induced ASS1 knockdown sensitized PDAC cells to the inhibitory effect of the combination of Arg deprivation and panobinostat on cell proliferation and viability (Figure 5B, Figure 5C, Supplementary Figure S2B). ASS1 knockdown alone did not affect PDAC cell proliferation (Figures 5B and 5C). In a panel of naturally existing ASS1-low PDAC cell lines, the combination of Arg deprivation and panobinostat showed significant growth inhibition in both anchorage-dependent monoculture (Figure 5D) and anchorage-independent PDAC cell culture models (Figure 5E, Supplementary Figure S3A and S3B). In ASS1-high PDAC cells, on the other hand, Arg deprivation had a smaller impact on enhancing the potency of HDAC inhibition (Figure 5D). In addition, Arg deprivation alone did not inhibit the growth of immortal human pancreatic duct epithelial (HPDE) cells, which have a near-normal genotype and phenotype 29, and Arg deprivation did not enhance the growth inhibitory effect of panobinostat (Figure 5F). Our results demonstrate that low ASS1 expression renders PDAC cells highly sensitive to the combination of Arg deprivation and panobinostat.

### HDAC inhibition induces DNA damage and apoptosis in cell cycle S-phase-arrested ASS1-low PDAC cells under arginine deprivation

**Figure 6:**
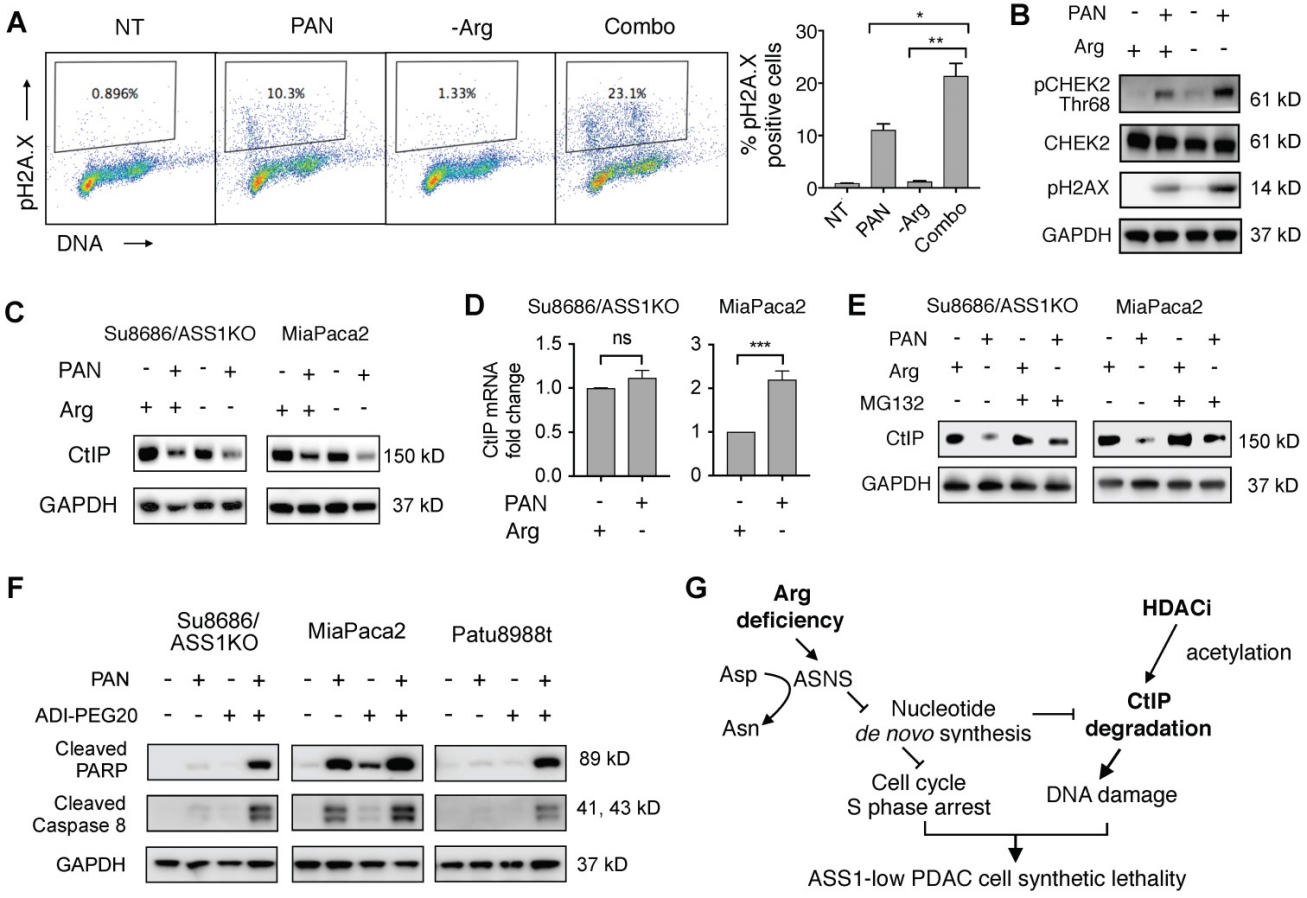
** HDAC inhibition induces DNA damage and causes synthetic lethality in PDAC cells arrested in S-phase by Arg deprivation.** (**A**) Flow cytometry analyses of DNA content and DNA damage marker pH2A.X in MiaPaca2 cells after 24 h treatment (n=2). (**B**) Western blot analysis for DNA damage markers in Miapaca2 cells after 24 h treatment. (**C**) Western blot analysis for CtIP after 24 h treatment with Panobinostat (PAN) and/or arginine (Arg) deprivation. (**D**) CtIP mRNA levels were quantified by qRT-PCR after 24 h treatment of PAN and Arg deprivation. (**E**) Proteasome inhibition rescued CtIP protein levels reduced by the combination of PAN and Arg deprivation. Cells were treated with PAN and Arg deprivation for 24 h. During the last 8 h, MG132 was added to cell culture. (**F**) Western blot analysis for apoptosis markers after 24 h treatment with Panobinostat and/or ADI-PEG20 in MiaPaca2 cells. (**G**) Schematic of mechanism for synergistic cytotoxicity of arginine deprivation and HDAC inhibition in ASS1-low PDAC. | Panobinostat: 25 nM (MiaPaca2), 50 nM (ASS1 KO, Patu8988t); ADI-PEG20: 0.5 µg/mL. NT = no treatment. * p<0.05, ** p<0.01, *** p<0.001.

We observed that the combination of HDAC inhibition and Arg deprivation induced substantially higher levels of DNA damage (21.4% pH2A.X positive) compared to single agent effects of HDAC inhibition (11.1% pH2A.X positive) or Arg deprivation (1.2% pH2A.X positive) in ASS1-low MiaPaca2 PDAC cells (Figure 6A). Similar to the changes in pH2A.X levels, the effect of HDAC inhibition on triggering DNA damage response signaling determined by pCHEK2 (Thr68) induction was enhanced by Arg deprivation (Figure 6B). An essential member of the homologous recombination DNA repair pathway, C-terminal-binding protein interacting protein (CtIP) is degraded by both HDAC inhibition (which leads to increased acetylation of CtIP in yeast cells 30) and by nucleotide insufficiency 31. Therefore, we hypothesized that HDAC inhibition and Arg depletion would additively induce CtIP degradation in human ASS1-low PDAC cells, leading to synergistic DNA damage and cell killing. To test this hypothesis, we first examined CtIP protein levels in both ASS1-deficient Su8686 PDAC cells and ASS1-low MiaPaca2 PDAC cells with HDAC inhibition and Arg depletion. We observed that HDAC inhibition substantially reduced CtIP protein levels, which were further reduced by Arg deprivation (Figure 6C). We also observed that HDAC inhibition and Arg depletion did not significantly affect CtIP mRNA levels in ASS1-deficient Su8686 cells, and upregulated CtIP mRNA levels in MiaPaca2 cells is likely secondary to transcriptional compensation for the reduction in CtIP protein levels (Figure 6D). Furthermore, CtIP protein levels reduced by HDAC inhibition and Arg depletion were rescued by a proteasome inhibitor MG132 (Figure 6E). Taken together, our results indicate that HDAC inhibition and Arg depletion collaboratively triggered DNA damage through the induction of CtIP protein degradation.

Cells in S-phase are most sensitive to DNA damage compared to other cell cycle phases 32-35. We therefore hypothesized that an increased S-phase cell population by Arg deprivation (Figure 3G) may further sensitize ASS1-low PDAC cells to DNA damage induced by HDAC inhibition and Arg deprivation, contributing to their synergistic effects. Consistent with our hypothesis, HDAC inhibition and Arg deprivation collaboratively induced higher levels of apoptosis compared to single agents in PDAC cells with low ASS1 expression (Figure 6D).

Our findings indicate that Arg-deprivation and HDAC inhibition in ASS1-low PDAC cells collaboratively cause CtIP degradation and trigger DNA damage while the increased S-phase cell population by Arg-deprivation sensitizes ASS1-low PDAC cells to DNA damage, with the end result being cellular apoptosis (Figure 6E).

### The combination of pharmacological arginine deprivation and panobinostat inhibits proliferation of ASS1-low PDAC cells in *in vitro* and *in vivo* tumor-like PDAC models

**Figure 7:**
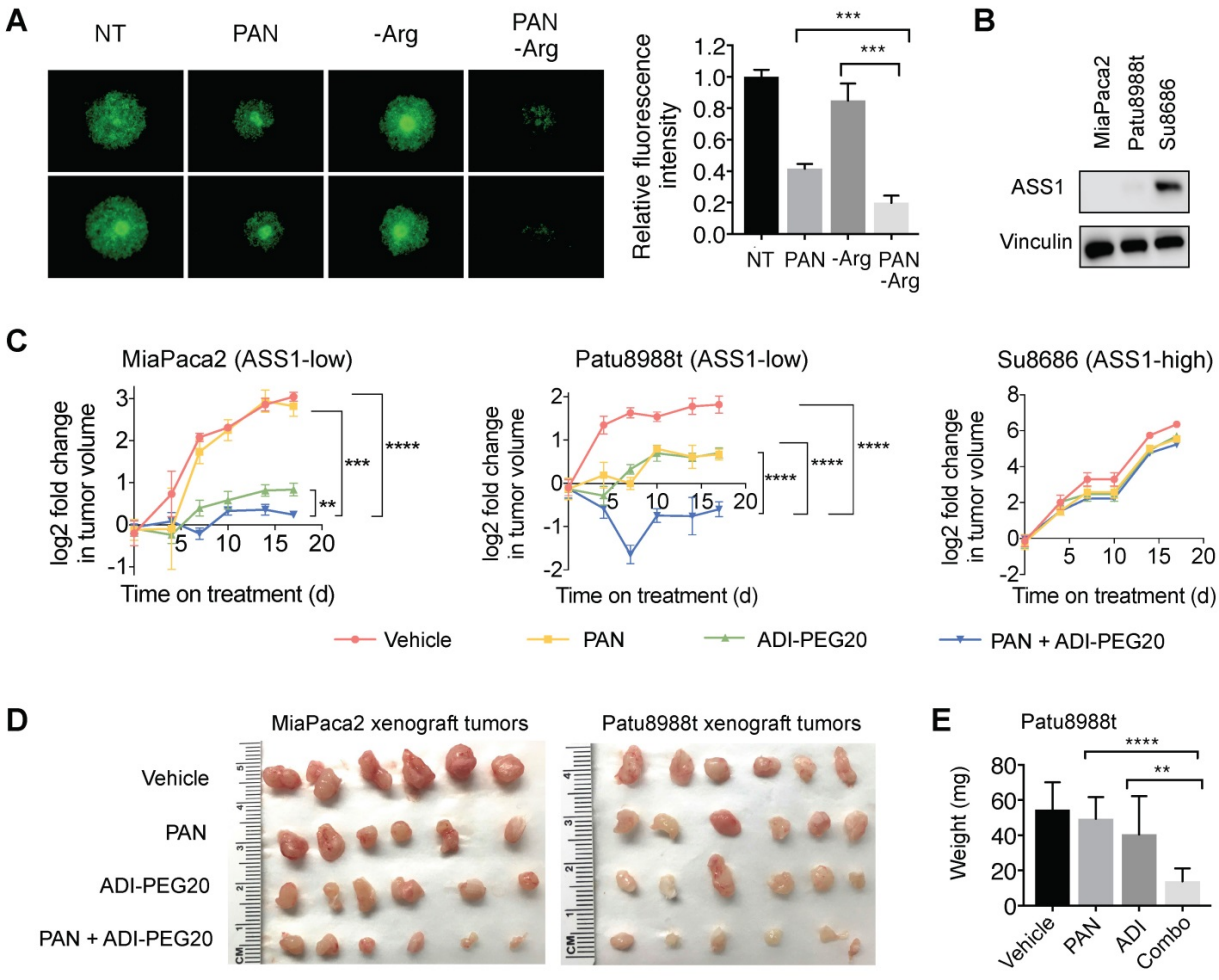
** Arg deprivation and PAN synergistically inhibit growth of ASS1-low tumor-like PDAC models *in vitro* and *in vivo*.** (**A**) Miapaca2-GFP cells co-cultured with PDAC cancer associated fibroblasts in anchorage-independent cell culture. Left, representative green fluorescence images taken after 5-day treatment. Right, quantified green fluorescence intensity. (**B**) ASS1 protein expression in indicated PDAC models. (**C**) Xenograft MiaPaca2, Patu8988t, and Su8686 tumor growth ± PAN ± ADI-PEG20. (**D**) MiaPaca2 and Patu8988t tumor images at explant**.** (**E**) Patu8988t tumor weights at explant.**ㅣ** RPMI media without Arg and Lys, supplemented with 0.3 mM Lys and 1 mM Cit ± 1 mM Arg. PAN: 25 nM (Panc1005, MiaPaca2), 50 nM (Panc0203, Patu8988t), 10 mg/kg (*in vivo*); ADI-PEG20: 5 IU. NT, Not treated. * p<0.05, ** p<0.01, *** p<0.001, **** p<0.0001.

Previous studies have shown that CAFs, which are abundantly present in the PDAC tumor microenvironment, can support cancer cell proliferation, migration, and invasion, and augment cancer cells' development of resistance to treatments 36-38. We used an established organotypic model 23, 36 where ASS1-low MiaPaca2 cells were co-cultured with PDAC cancer-associated fibroblasts (CAFs). In our co-culture PDAC organotypic model, we found that the synergistic growth inhibition of panobinostat and Arg deprivation remained potent in the presence of CAFs (Figure 7A).

To further evaluate the synergy of HDAC inhibition and Arg deprivation, we used two representative ASS1-low PDAC xenograft models (MiaPaca2 and Patu8988t) and a representative ASS1-high PDAC xenograft model (Su8686) (Figure 7B). The combination of panobinostat and ADI-PEG20 significantly and synergistically slowed the growth of ASS1-low MiaPaca2 and Patu8988t tumors, whereas a marginal synergistic effect was observed on the growth of the ASS1-high Su8686 tumors (Figure 7C). The combination of panobinostat and ADI-PEG20 reduced tumor growth by 75% in MiaPaca2 tumors and by 67% in Patu8988t tumors (Figure 7C). Drug treatments were well-tolerated by mice with less than 10% body weight loss throughout the treatment period. At the end of treatment, tumors were harvested (Figure 7d) and weighed to confirm the synergistic effect of panobinostat and ADI-PEG20 on suppressing ASS1-low, but not ASS1-high, PDAC tumor growth (Figure 7E). Taken together, our findings provide a rational, promising translational strategy for treating ASS1-low PDAC tumors.

## Discussion

Studies have shown that reduced ASS1 expression is associated with aggressive tumor behavior and worse prognosis in various cancers including hepatocellular carcinoma, myxofibrosarcomas, bladder cancer, and nasopharyngeal carcinoma 14, 15, 39, 40. In this study, we found that low ASS1 expression was associated with worse overall survival and higher tumor grade on a large and well-annotated tissue microarray of 138 early stage PDACs that underwent surgical resection at our institution. Our findings also showed that ASS1 expression was an independent predictor of overall survival in our cohort after multivariate analysis. Our findings expand previously reported observations that low ASS1 expression is significantly associated with worse prognosis, tumor size greater than 2 cm, positive margin, and lymph node status 41. Mechanistically, loss of ASS1 increases the flux of Asp in *de novo* nucleotide biosynthesis 16, which, in turn, could interfere with the effects of 5-FU and gemcitabine 42, the backbone of standard-of-care therapy in PDAC. These findings suggest an urgent need for further research into finding an effective treatment to target this aggressive cancer subset.

Arg deprivation therapy is a promising strategy to target ASS1-low cancers because, by delivering PEGylated arginine deiminase (ADI-PEG20, Polaris Pharmaceuticals), it degrades the extracellular Arg that ASS1-low tumors with low intracellular synthetic capacity depend on for survival and progression. However, resistance mechanisms to Arg deprivation therapy have been reported, suggesting that it is inadequate as a monotherapy for cancer treatment 43. As such, combination therapies with Arg deprivation have been studied in various cancers, in both pre-clinical settings and clinical trials 11. In PDAC, pre-clinical studies have shown that Arg deprivation therapy increased the cytotoxicity of gemcitabine 44, 45. In a search for an optimal translational combination regimen with Arg deprivation for ASS1-low PDAC, we performed an unbiased screen of FDA-approved oncology drugs and made the novel observation that HDAC inhibitors were synergistic with Arg deprivation.

HDAC inhibitors are an important class of oncology drugs. Multiple strategies have been reported to improve their anti-cancer efficacy 36, 46-48. Panobinostat, a clinical HDAC inhibitor used as therapy for multiple myeloma, has been studied extensively in PDAC in pre-clinical studies and clinical trials. Several mechanisms of cytotoxicity have been reported in PDAC including induction of apoptosis, autophagy, and cell cycle arrest 49. We found that panobinostat treatment reduced the protein level of a key DNA repair enzyme, CtIP, which was further reduced by Arg depletion in ASS1-low PDAC cells. It has been reported in yeast cells that HDAC inhibition induced yeast Sae2 (human CtIP) acetylation and degradation 30. Consistent with these previous observations, our findings provided the first evidence in human cells that HDAC inhibition results in CtIP degradation. Furthermore, we found that Arg deprivation in ASS1-low PDAC cells upregulated the expression of ASNS which redirects Asp from *de novo* nucleotide biosynthesis and causes nucleotide insufficiency. Consistent with our findings, previous observations in breast cancer cells showed that Arg starvation in cell culture induced ASNS expression 50. In addition, Qu et al reported that knockdown of phosphoglycerate mutase 1 (PGAM1), an important glycolytic enzyme in cancer cells, accelerated CtIP degradation through deprivation of the intracellular deoxyribonucleotide triphosphate pool 31, which is consistent with our observations.

DNA damage induction is a critical mechanism for anti-cancer therapies 51, 52. Throughout the cell cycle, the S-phase cell population is most vulnerable to DNA damage 32-35. This occurs for two reasons. First, the chromatin structure of DNA in S-phase is partially unwrapped to be more accessible to DNA replication machinery; therefore, the chromosomes are unprotected and more susceptible to DNA damage induced by both external and internal agents. Second, transcription, replication, and DNA repair co-occur in S-phase and thus the likelihood of conflict between these machineries is elevated. Nucleotide insufficiency is an established cause of cell cycle S-phase arrest 23, 24. Here, we found that Arg deprivation induced ASNS-mediated nucleotide insufficiency and triggered cell cycle S-phase arrest, thus sensitizing ASS1-low PDAC cells to DNA damage caused by HDAC inhibition.

Interestingly, MiaPaca2 and Patu8988t are two naturally ASS1-low PDAC cell lines, but they do not share the same changes in apoptotic protein expression after treatment with panobinostat or ADI-PEG20 when each drug is applied alone (Figure 6F). This difference may be associated with the differentiation status as well as their site of derivation. MiaPaca2 is a poorly differentiated cell line established from primary PDAC 53, whereas Patu8988t is a well differentiated cell line derived from liver metastases 54. In addition, the ASS1KO Su8686 isogenic cell line exhibited a similar pattern of apoptotic protein expression compared to that of Patu8988t (Figure 6F). Similarly, the parental cell line Su8686 is more differentiated than MiaPaca2 and was isolated from liver metastases 53. Therefore, the difference in the apoptotic protein expression pattern induced by panobinostat and ADI-PEG20 could be associated with the differentiation status as well as derivation site.

ASS1-low PDAC tumors are highly aggressive with a poor prognosis, but our findings provide the mechanistic basis for a rational translational therapeutic approach for this subset of PDAC tumors with a combination of pharmacological Arg deprivation agents and HDAC inhibitors.

## Supplementary Material

Supplementary figures and tables.
