# Supplementary material for: Histone deacetylase inhibition is synthetically lethal with arginine deprivation in pancreatic cancers with low argininosuccinate synthetase 1 expression
Source: Theranostics. 2020 Jan 1;10(2):829–40. doi: 10.7150/thno.40195 (PMC6929997; doi:10.7150/thno.40195)
Supplement: Supplementary file 1 — Supplementary figures and tables. [file thnov10p0829s1.pdf]

SUPPLEMENTAL MATERIALS AND METHODS

Antibodies.

ASS1 antibody (sc-365475) was purchased from Santa Cruz biotechnology. GAPDH (#5174), CtIP (#9201) pH2A.X (#9718), pCHEK2 (#2661), CHEK2 (#2662), Cleaved PARP (#5625), Cleaved Caspase 8 (#9496), Vinculin (#13901) were from Cell Signaling Technology. pH2AX S139-FITC (05-636) antibody was purchased from Millipore, and ASNS antibody (14681-1-AP) was purchased from Proteintech.

Arginine Deprivation.

Arginine deprivation experiments were performed using RPMI 1640 Medium for SILAC (ThermoFisher Scientific #88365) with addition of 10% dialyzed FBS, 0.04 g/L L-Lysine (Sigma), and 1 mM L-Citrulline (Sigma). L-Arginine (Sigma) was added at 1 mM concentration for appropriate conditions.

Drugs.

ADI-PEG20 was provided by Polaris Pharmaceuticals. The following drugs were suspended in DMSO (Sigma): Vorinostat (Biotang), Osimertinib (MedChemExpress), Topotecan (SelleckChem), Bosutinib (MedChemExpress), Permetrexed (SelleckChem), Lenalidomide (MedChemExpress), Belinostat (MedChemExpress), Cytarabine (SelleckChem), Panobinostat (Selleckchem), Afatinib (MedChemexpress).

Proliferation assay.

Cells were plated in 96-well plates (1,000 cells/well). Treatments were added 24 h after plating. After 72 h treatment, cell mass was measured using CellTiter-Glo reagent (Promega) and analyzed by Synergy H1 Hybrid Reader (Biotek). For anchorage-independent culture, PDAC cells were grown on tissue-culture plates treated with poly(2-hydroxyethyl methacrylate (poly-

HEMA) (Sigma). Briefly, Poly-HEMA was dissolved at 20 mg/mL concentration in 95% Ethanol and added to the plate to fully cover growth area and dried overnight.

Colony formation assay.

Cells were seeded in 12-well plates at 50,000 cells/well in duplicate. After 24 hour treatment, media was replaced and cells were incubated for 7 days. Cells were washed with PBS and fixed in 4% PFA. Cells were then stained with 0.1% Crystal Violet (Sigma) for 30 minutes and washed with water.

***In vitro* tumor cell-fibroblast 3D co-culture.**

$1x10^3$ MiaPaca2-GFP cells were plated in U-bottom black-walled 96-well ultralow attachment plates (Corning). After 48 h, $8x10^3$ PDAC cancer associated fibroblasts were added (day 0). 72 h later spheroids were formed and treatment was initiated. Daily fluorescence readings were taken using a blue optical kit (Ex 490nm/Em 510-570nm) on a Modulus II Microplate Multimode Reader. Images were taken using a CX41 Inverted Microscope with a DP26 Digital Camera (Olympus).

Generation of ASS1 knockout isogenic cells.

Four sgRNA sites were designed for the exon4 of the ASS1 gene. The recombinant plasmids of Lentiviral vector2-ASS1-sgRNA were constructed. The constructed vector were transfected into SU8686 cells. then 24 hr later cells were treated with puromycin for 3 days. The two gRNAs (#1: TATGTGTCCCACGGCGCCAC and #3: ATACTTGGCCCCCTCCCGCT) with best knockout effect of ASS1 gene in SU8686 cells were selected. Single cell cloning was performed with limited dilution of cells into 96-well plates. At least 2 single clones of each gRNA were selected for knockout confirmation with Western blot.

Generation of ASS1 shRNA Knockdown cells.

For inducible ASS1 knockdown, ASS1 shRNA oligonucleotides (shASS1 5’-CACCGCTATGACGTCATTGCCTATCTTCAAGAGAGATAGGCAATGACGTCATAGC-3’ and 5’-

AAAAGCTATGACGTCATTGCCTATCTCTCTTGAAGATAGGCAATGACGTCATAGC-3’; and shASS1 5’-CACCGGATGCCTGAATTCTACAACCTTCAAGAGAGGTTGTAGAATTCAGGCATCC-3’ and 5’-AAAAGGATGCCTGAATTCTACAACCTCTCTTGAAGGTTGTAGAATTCAGGCATCC-3’) were annealed and ligated into pENTR/H1/TO vector (Invitrogen #K4920-00) following BLOCK-iT Inducible H1 RNAi Entry Vector Kit manual. Resulting shRNA constructs were recombined into pLentipuro/BLOCK-iT-DEST using Gateway LR Clonase II (Invitrogen #11791-020). Recombinant lentiviruses were packaged in 293T cells by co-transfecting each of lentivirus plasmid with expression vectors containing the gag/pol, rev and vsvg genes. Lentivirus was harvested 48 hours after transfection and added to subconfluent Su8686 cells with polybrene for 16 hours. Cells were selected in puromycin for 1 week. Doxycycline induction of knockdown is controlled by the Tet repressor (TetR) protein expressed from the pLenti0.3/EF/GW/IVS-Kozak-TetR-P2A-Bsd vector. Knockdown was induced with 100 ng/mL doxycycline.

Western blotting.

Cells were lysed in cold RIPA lysis buffer with protease and phosphatase inhibitors (Thermo Scientific). Protein extracts were resolved on SDS-PAGE and then electrotransferred to Immun-Blot Nitrocellulose membrane (Bio-Rad, Hercules, CA, USA). After blocking in 5% milk, membranes were incubated in primary antibody solution at 4 ℃ overnight, and then with horseradish peroxidase (HRP)-conjugated secondary antibody solution at room temperature for 1h. Blots were developed using Pierce ECL Substrate (Thermo Scientific, Rockford, IL) and imaged on the LI-COR Odyssey imaging system.

RNA-Seq.

Samples were prepared in triplicate. Cells were trypsinized and collected on ice after 24 h treatment for RNA extraction and analysis. Libraries for RNA-Seq were prepared with KAPA Stranded mRNA Kit. The workflow consisted of mRNA capture, cDNA generation, and end

repair to generate blunt ends, A-tailing, adaptor ligation and PCR amplification. The data was sequenced on Illumina HiSeq 3000 for a single-read 50 bp run. Data quality check was performed on Illumina SAV. The reads were mapped to the latest UCSC transcript set using Bowtie2 version 2.1.0 (1) and the gene expression level was estimated using RSEM v1.2.15 (2). Normalized gene expression data were fed into Ingenuity Pathway Analysis (3).

Sample preparation for LC-MS/MS-MRM analysis of nucleotide pools and incorporation of labeled nucleotides into newly replicated DNA and RNA.

For glucose labeling experiments, cells were cultured in DMEM without glucose or glutamine + 10% dialyzed FBS (Gibco) and 4mM glutamine (Gibco) containing [U-$^{13}C_6$]glucose (Sigma-Aldrich, 389374) at 1g/L. Prior to sample collection, labeled media was aspirated and cells were washed and collected by trypsinization. Cells were pelleted then lysed with 10µL of 10% trifluoroacetic acid with internal standards (1µM [$^{15}N_3$]dCMP and [$^{15}N_3$]dCTP, Silantes # 122303802 and # 120303802, respectively) vortexed for 30s, and incubated on ice for 10m. 40 µL of 1 M ammonium acetate, pH=10, with the same internal standard added and the samples vortexed again for 30s, and centrifuged to remove insoluble cellular material. The supernatants (~40µL) were transferred into HPLC injector vials. Stock solutions (10 mM) of dCTP, dATP, dGTP, and dTTP (Sigma Aldrich) used to generate reference standards, and were processed prior to dNTP as metrics of LC-MS/MS-MRM signal and proportionally compared to previous runs to account for signal variation.

Genomic DNA was extracted using the Quick-gDNA MiniPrep kit (Zymo Research, D3021) and hydrolyzed to nucleosides using the DNA Degradase Plus kit (Zymo Research, E2021). In the final step of DNA extraction, 50 µL of water was used to elute DNA into 1.5mL microcentrifuge tubes. A nuclease solution (5µL; 10X buffer/DNA Degradase Plus™/water, 2.5/1/1.5, v/v/v) was added to 20 µL of the eluted genomic DNA or RNA in an HPLC injector vial.

Targeted LC-MS/MS-MRM assays.

For free nucleotide analysis, a modified version of the previously reported method (4) was used in which each dNTP lysate sample (15 µL) was injected directly onto the Hypercarb column equilibrated in solvent A (5mM hexylamine and 0.5% diethylamine, v/v, pH 10.0) and eluted (200 µL/min) with an increasing concentration of solvent B (acetonitrile/water, 50/50, v/v) at the following min/%B/flow rates (µL/min): 0/5/200, 20/100/200, 20.50/100/600, 22.50/100/600, 23/5/600, 25/5/600, 25.5/5/200, 28/5/200. The effluent from the column was directed to the Agilent Jet Stream ion source connected to the triple quadrupole mass spectrometer (Agilent 6460) operating in the multiple reaction monitoring (MRM) mode using previously optimized settings. The peak areas for each nucleosides and nucleotides (precursor→fragment ion transitions) at predetermined retention times were recorded using software supplied by the instrument manufacturer (Agilent MassHunter).

For RNA and DNA analyses, an aliquot of the hydrolyzed sample (15 µL) was injected onto a porous graphitic carbon column (Thermo Fisher Scientific Hypercarb, 100 x 2.1mm, 5µm particle size) equilibrated in solvent A (water 0.1% formic acid, v/v) and eluted (200 µL/min) with an increasing concentration of solvent B (acetonitrile 0.1% formic acid, v/v) using min/%B/flow rates (µL/min) as follows: 0/0/700, 3.5/70/700, 4.5/70/700, 5/0/700, 7/0/7.

Quantification.

The areas for nucleotide measurements were obtained from extracted ion chromatograms of MRM ion transitions. These measurements were normalized from the spiked internal standards ([^{15}N3]dCMP and [^{15}N3]dCTP). Nucleotide data were normalized to cell number and displayed as relative amount per cell compared to untreated. For DNA, the areas for the hydrolyzed labeled nucleosides were obtained from extracted ion chromatograms of MRM ion transitions, and normalized to total ion current at that retention time.

pH2A.X assay.

Cells were harvested, fixed, permeabilized with cytofix/cytoperm (BD biosciences, #554722) for 15 min on ice, prior to staining with a phospho-Histone H2A.X (Ser139) antibody conjugated to fluorochrome FITC (EMD Millipore, #05- 636, 1:800 dilutions in perm/wash) for 20 min in the dark at room temperature. Subsequently, cells were washed and stained with 0.5 mL of DAPI (Invitrogen, #D1306) for DNA content before data acquisition by flow cytometry.

5-ethynyl2-deoxyuridine (EdU) pulse-chase cell cycle profiling.

MiaPaca2 cells were plated at 0.3 x 10^6 cells/mL, pulsed with 10 μM EdU (Invitrogen) for 2 h, washed twice with PBS, and released in fresh media containing 5 μM deoxyribonucleosides. Cells were collected 4 h following release in fresh media, and then fixed with 4% paraformaldehyde and permeabilized using saponin perm/wash reagent (Invitrogen), and then stained with azide-Alexa Fluor 647 by Click reaction (Invitrogen; Click-iT EdU Flow cytometry kit, #C10634). The total DNA content was assessed by staining with FxCycle-Violet (Invitrogen, #F10347) at 1 μg/mL final concentration. Flow cytometry data were acquired on five-laser LSRII cytometers (BD), and analyzed using FlowJo software (Tree Star). The cell cycle durations were calculated using equations for multiple time-point measurements according to previously published methods (5).

REFERENCES

1. Langmead B, Salzberg SL. Fast gapped-read alignment with Bowtie 2. Nat Methods. 2012; 9: 357-9
2. Li B, Dewey CN. RSEM: accurate transcript quantification from RNA-Seq data with or without a reference genome. BMC Bioinformatics. 2011; 12: 323
3. Kramer A, Green J, Pollard J, Jr., Tugendreich S. Causal analysis approaches in Ingenuity Pathway Analysis. Bioinformatics. 2014; 30: 523-30
4. Cohen S, Megherbi M, Jordheim LP, Lefebvre I, Perigaud C, Dumontet C*, et al.* Simultaneous analysis of eight nucleoside triphosphates in cell lines by liquid chromatography coupled with tandem mass spectrometry. J Chromatogr B Analyt Technol Biomed Life Sci. 2009; 877: 3831-40
5. Terry NH, White RA. Flow cytometry after bromodeoxyuridine labeling to measure S and G2+M phase durations plus doubling times in vitro and in vivo. Nat Protoc. 2006; 1: 859-69

Supplementary Table S1 | Primers for qRT-PCR gene expression analysis.

Gene symbol	Primer	Primer sequence (5' to 3')
RBBP8 (gene encoding CtIP)	Forward	GCTTGACTTTGTCTTGTTTATCACC
	Reverse	AGAGTATGAATCCTGTTTGGCA
ACTB	Forward	CTGGCTGCCTCCACCCACTCCCA
	Reverse	CCATCCTGGCCTCGCTGTCCAC

Supplementary Table S2 | Clinicopathologic characteristics of 138 PDAC patient samples by ASS1 expression.

Characteristics	ASS1-low (n=69)	ASS1-high (n=69)	*p* value
**Gender**			0.609
Female	31	35	
Male	38	34	
**Age**			0.150
<60	28	19	
>60	41	50	
**Tumor grade**			**0.016**
Low	31	46	
High	38	23	
**Tumor size**			0.489
<3cm	43	38	
>3cm	26	31	
**Margin status**			0.274
Negative	64	59	
Positive	5	10	
**Tumor stage**			1.00
pT1 or pT2	40	40	
pT3	29	29	
**Lymph node status**			1.00
Negative	33	36	
Positive	34	35	

Supplementary Table S3 | Low ASS1 expression is an independent predictor of survival in a multivariate Cox regression model with other significant clinicopathologic variables.

Variable	Comparison	Hazard Ratio (95% Confidence Interval)	*P*-value
**ASS1**	Low vs. High	1.54 (1.11-2.47)	0.014
**Sex**	Female vs. Male	1.42 (0.97-2.10)	0.074
**Grade**	G1-G2 vs. G3-G4	0.50 (0.33-0.77)	0.002
**pN**	pN0 vs. pN1	0.54 (0.36-0.80)	0.003
**pT**	pT1-T2 vs. pT3	0.64 (0.42-0.98)	0.038

Hazard ratio >1 indicates greater risk of death for the first variable indicated. All covariates retained in the model after backward selection using Akaike Information Criterion are shown here.

Supplementary Table S4 | List of 133 FDA-approved oncology drugs.

Allopurinol	Mercaptopurine	Enzalutamide	Omacetaxine mepessucinate
Dacarbazine	Altretamine	Romidepsin	Etoposide
Azacitidine	Uracil mustard	Doxorubicin hydrochloride	Lapatinib
Procarbazine hydrochloride	Cisplatin	Plicamycin	Paclitaxel
Gemcitabine hydrochloride	Letrozole	Vinorelbine tartrate	Everolimus
Chlorambucil	Clofarabine	Vinblastine sulfate	Plerixafor
Megestrol acetate	Fludarabine phosphate	Irinotecan hydrochlrodie	Idarubicin hydrochloride
Niraparib hydrochloride	Mitoxantrone	Dabrafenib mesylate	Cobimetinib
Lenvatinib	Idelalisib	Amifostine	Ceritinib
Topotecan hydrochloride	Raloxifene	Erismodegib	Sirolimus
Fluorouracil	Methlorethamine hydrochloride	Ixazomib citrate	Trametinib
Arsenic trioxide	Floxuridine	Tamoxifen citrate	Triethylenemelamine
Decitabine	Cytarabine hydrochloride	Vincristine sulfate	Ixabepilone
Streptozocin	Tretinoin	Docetaxel	Daunorubicin hydrochloride
Vorinostat	Lenalidomide	Venetoclax	Dactinomycin
Melphalan hydrochloride	Belinostat	Pazopanib hydrochloride	Valrubicin
Bendamustine hydrochloride	Capecitabine	Vemurafenib	Carfizomib
Bortezomib	Gefitinib	Ponatinib	Zoledronic acid
Vismodegib	Ibrutinib	Epirubicin hydrochloride	Crizotinib
Dasatinib	Afatimib	Teniposide	Sorafenib
Hydroxyurea	Thiotepa	Temsirolimus	Olaparib
Temozolomide	Methoxsalen	Imiquimod	Uridine triacetate
Carmustine	Thalidomide	Nilotinib	Permetrexed, Disodium salt, Heptahydrate
Cladribine	Dexrazoxane	Regorafenib	Sunitinib
Exemestane	Nelarabine	Cabozantinib	Oxaliplatin
Mitomycin	Panobinostat	Estramustine phosphate sodium	Methotrexate
Carboplatin	Celecoxib	Bleomycin sulfate	Pipobroman
Axitinib	Erlotinib hydrochloride	Fulvestrant	Mitotane
Rucaparib phosphate	Ribociclib	Palbociclib	Vandetanib
Imatinib	Pralatrexate	Alcetinib	Pomalidomide
Thioguanine	Aminoleviulinic acid hydrochloride	Osimertinib	Anastrozole
Busulfan	Lomustine	Bosutinib	
Cyclophosphamide	Trifluridine	Cabazitaxel	
Ifosfamide	Pentostatin	Abiraterone	

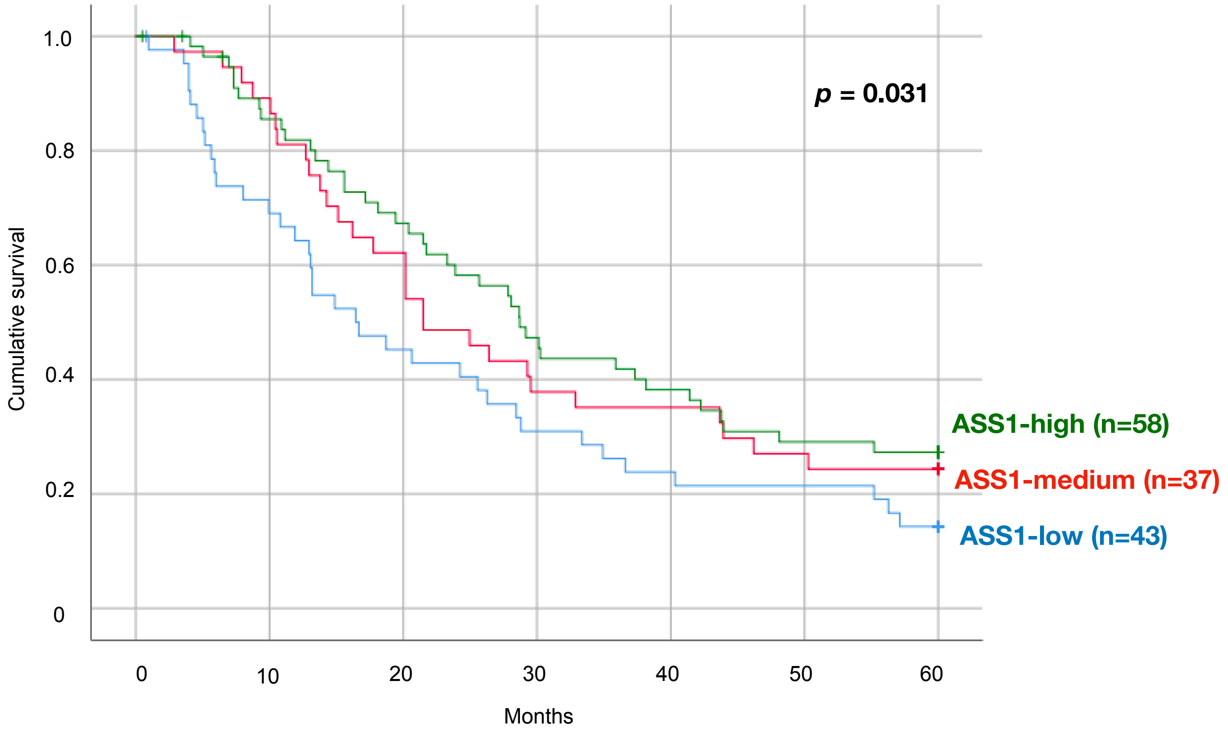

Supplementary Figure S1 | Low ASS1 expression is associated with worse prognosis in PDAC patients. Kaplan-Meier analysis of 138 PDAC patients divided into three groups by ASS1 expression histoscore.

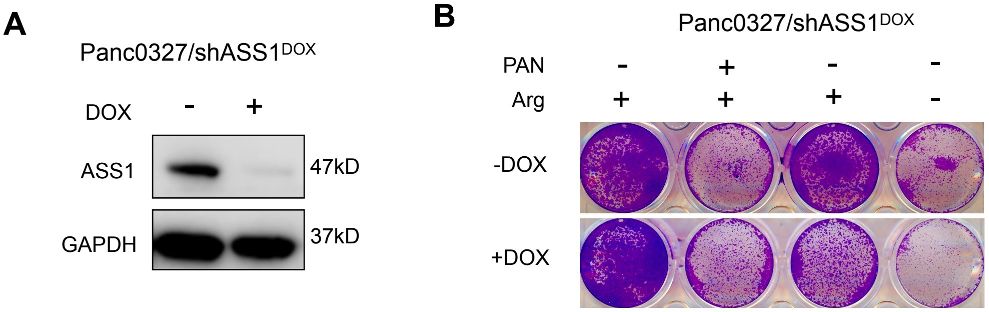

Supplementary Figure S2 | Knockdown of ASS1 expression causes increased sensitivity to PAN and Arg deprivation in ASS1-high Panc0327 cells. (**A**) DOX-inducible ASS1 shRNA knockdown in Panc0327 cells. (**B**) Colony formation assay after 7 d treatment with +/- DOX +/- PAN +/- Arg in Panc0327 ASS1 KD cells. | RPMI media without Arg and Lys, supplemented with 0.3 mM Lys and 1 mM Cit ± 1 mM Arg. PAN: 50 nM; DOX: 100 ng/mL.

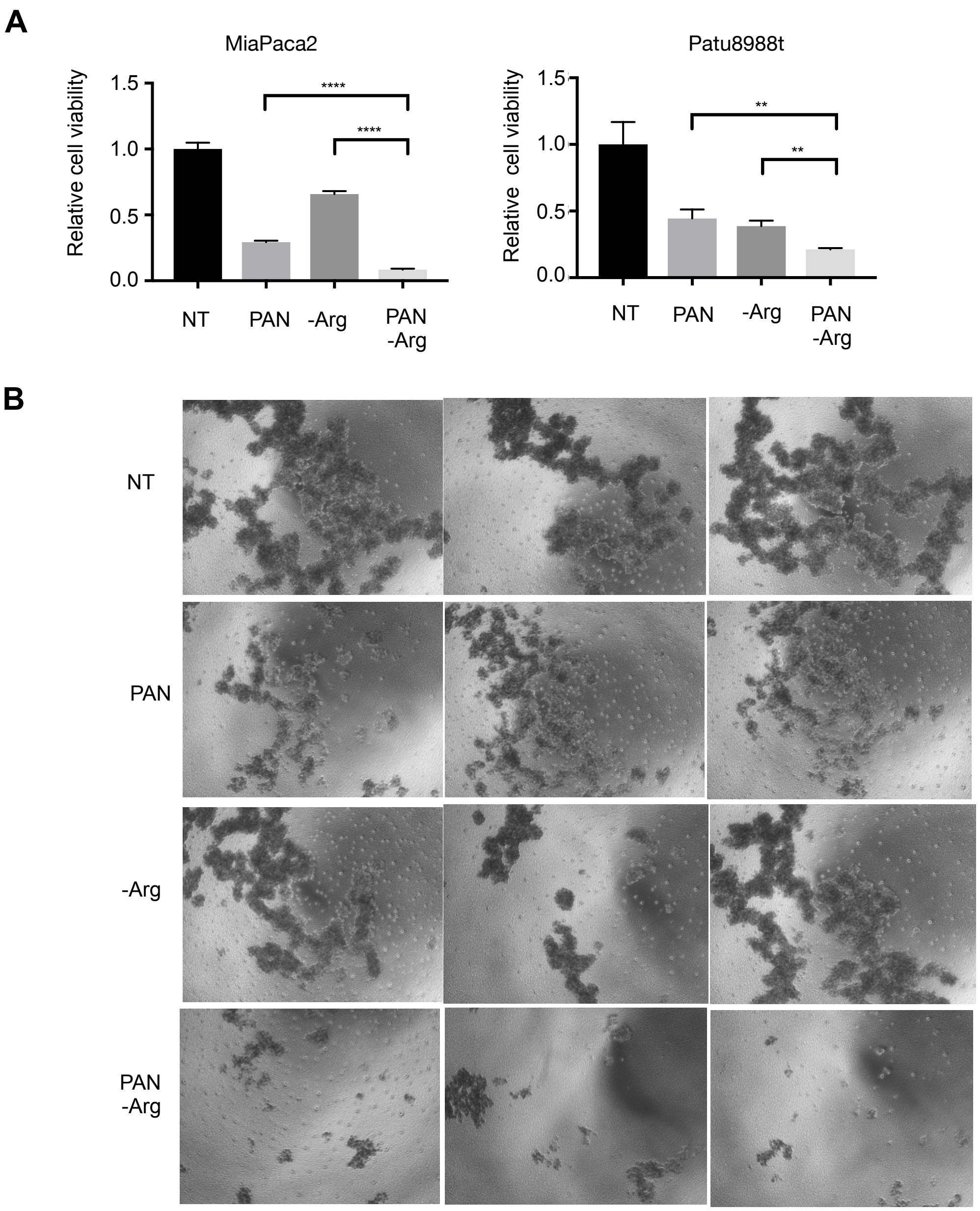

Supplementary Figure S3 | Arg deprivation and PAN synergistically inhibit growth of ASS1-low PDAC cells in anchorage-independent cell culture model. (**A**) Viability of ASS1-low PDAC cells with 72 h PAN and Arg deprivation treatments in anchorage-independent cell culture model. (**B**) MiaPaca2 in anchorage-independent cell culture with indicated treatments for 5 days. | RPMI media without Arg and Lys, supplemented with 0.3 mM Lys and 1 mM Cit ± 1 mM Arg. PAN: 25 nM (MiaPaca2), 50 nM (Patu8988t). ** $p<0.01$, *** $p<0.001$, **** $p<0.0001$.
